# Clinical benefits of adding dexamethasone to local infiltration analgesia for simultaneous bilateral total hip or knee arthroplasty

**DOI:** 10.1186/s13018-024-04620-3

**Published:** 2024-03-01

**Authors:** Xiao-Dong Wang, Qing-Wei Meng, Fu-Shan Xue

**Affiliations:** 1 Department of Anesthesiology, Wei-Hai Municipal Hospital, Cheeloo College of Medicine, Shandong University, Weihai, Shandong, People’s Republic of China; 2 Department of Anesthesiology, Beijing Friendship Hospital, Capital Medical University, NO. 95 Yong-An Road, Xi-Cheng District, Beijing, 100050 People’s Republic of China

To the editor,

By conducting a randomized controlled double-blind trial of 105 patients who received simultaneous bilateral total hip or knee arthroplasty, Wang et al. [1] showed that the addition of dexamethasone to the local infiltration analgesia (LIA) mixture decreased total opioid consumption, frequency of using patient-controlled intravenous analgesia (PCIA) and the occurrence of postoperative nausea and vomiting. We sincerely congratulate the authors for publishing their work, but wish to present our questions on their methodology and results.

First, in sample size evaluation, the authors described that mean ± standard deviation for dose of morphine mg equivalent (MME) within 24 h postoperatively in the patients who underwent simultaneous bilateral total joint arthroplasty and did not use dexamethasone, was 78.6 ± 58.3 mg and an expected decrease of 30% in cumulative opioid consumption within 24 h postoperatively, i.e., a reduction of about 24 MME, was used as the effect size. This may not be a universally acknowledged effect size. For patients undergoing total hip or knee arthroplasty, the recommended minimal clinically important difference of cumulative opioid consumption for postoperative pain control in available literature actually is an absolute reduction of 10 mg intravenous morphine in 24 h [2]. The total opioid consumption during postoperative hospitalization of 3 days in this study was significantly decreased in patients receiving addition of dexamethasone, but the between-group difference in mean MME was 17.4. That is, average daily decrease in total opioid consumption only is 5.8 and does not exceed the recommended minimal clinically important difference. In this case, it is somewhat difficult for the reader to determine whether opioid-sparing efficacy with addition of dexamethasone to the LIA mixture in the patients receiving simultaneous bilateral total hip or knee arthroplasty is clinically significant. We believe that clarification of these issues would improve the transparency of this study design and help the interpretation of the results.

Second, other than LIA and nonsteroidal anti-inflammatory drugs, both PCIA with sufentanil and rescue analgesia with dezocine were also used for postoperative multimodal analgesia. However, the authors did not clearly state their target of postoperative pain control. According to the data of Table 1 and Figs. 2 and 3 of Wang et al.’ article [1], we noticed that mean postoperative pain visual analog scale (VAS) scores at resting during postoperative days 0–2 were more than 3 and exceeded preoperative levels. Even mean postoperative pain VAS scores during movement at postoperative days 0–2 were up to 4.9–6.1. These results indicate that most patients in the two groups had significant postoperative pain, especially during movement. Because significant postoperative pain can reduce patient’ early mobilization and rehabilitation exercise, the enhanced recovery after surgery practice for total hip or keen arthroplasty requires the use of a multimodal analgesia strategy to achieve a VAS score of 3 or less for patient comfort and rapid postoperative recovery [3]. We argue that inadequate postoperative pain control in this study may hinder the generalization of the findings into the current enhanced recovery after surgery practice for total hip or knee arthroplasty.

Finally, unlike other previous works assessing postoperative benefits of regional blocks [4, 5], this study did not evaluate patients’ satisfaction with postoperative pain control and their postoperative functional and recovery outcomes, such as times to start mobilization and rehabilitation exercise, low limb function, pain-related readmissions, quality of recovery, length of hospital stay and others. Thus, an important issue that is not addressed in this study is whether the addition of dexamethasone to the LIA mixture in the patients receiving simultaneous bilateral total hip or knee arthroplasty can be translated into clinical benefits of the enhanced recovery after surgery practice.

## Data Availability

Not applicable.
